# Astrocytes in schizophrenia

**DOI:** 10.1177/23982128211009148

**Published:** 2021-04-27

**Authors:** Tina Notter

**Affiliations:** Neuroscience and Mental Health Research Institute, Cardiff University, Cardiff, UK

**Keywords:** Astrocytes, schizophrenia, neurodevelopment, glutamate hypothesis

## Abstract

Schizophrenia is a severe and clinically heterogenous mental disorder affecting approximately 1% of the population worldwide. Despite tremendous achievements in the field of schizophrenia research, its precise aetiology remains elusive. Besides dysfunctional neuronal signalling, the pathophysiology of schizophrenia appears to involve molecular and functional abnormalities in glial cells, including astrocytes. This article provides a concise overview of the current evidence supporting altered astrocyte activity in schizophrenia, which ranges from findings obtained from post-mortem immunohistochemical analyses, genetic association studies and transcriptomic investigations, as well as from experimental investigations of astrocyte functions in animal models. Integrating the existing data from these research areas strongly suggests that astrocytes have the capacity to critically affect key neurodevelopmental and homeostatic processes pertaining to schizophrenia pathogenesis, including glutamatergic signalling, synaptogenesis, synaptic pruning and myelination. The further elucidation of astrocytes functions in health and disease may, therefore, offer new insights into how these glial cells contribute to abnormal brain development and functioning underlying this debilitating mental disorder.

## Introduction

Schizophrenia (SZ) is a devastating mental disorder with a worldwide prevalence of approximately 0.7%–1% (Owen et al., 2016). Core features of the clinical manifestation consist of positive symptoms (symptoms such as hallucinations, delusions, disorganised speech and thought), negative symptoms (a decrease or absence of normal behaviour related to emotion, motivation or interest) and cognitive symptoms (such as dysfunctions in working memory, verbal memory, attention and executive functioning) (Correll and Schooler, 2020; Owen et al., 2016). The clinical course, presentation and prognosis of SZ are highly heterogeneous, generally associated with multiple and hardly predictable relapses, and often manifest itself in disease chronicity (Lewis and Lieberman, 2000; Owen et al., 2016). The first episode of psychosis typically occurs during late adolescence or early adulthood (Owen et al., 2016; Sommer et al., 2016). Prior to the first episode, most but not all patients show an at-risk mental state (prodromal phase) (Addington and Heinssen, 2012; Lieberman et al., 2001), which in some cases is accompanied by cognitive and social deficits (Lewandowski et al., 2011). Upon full manifestation of SZ, patients are typically assigned to long-term pharmacological treatments, which are, however, estimated to be effective in only ~66% of the patients, whereas the remaining ~34% are treatment resistant (Patel et al., 2014; Potkin et al., 2020).

Despite the continuous research efforts, the aetiology of SZ remains largely elusive. SZ is now considered a neurodevelopmental disorder that involves complex interactions between multiple genetic and environmental susceptibility factors (Insel, 2010). Converging evidence from large-scale genetic studies suggests SZ has a polygenetic component, whereby increased disease risk is conferred by an accumulation of common and rare variants with relatively low effect sizes (Owen et al., 2016). In addition to its genetic basis, SZ has also been associated with numerous environmental risk factors, including prenatal exposure to infectious or non-infectious maternal immune activation (MIA), childhood stress and trauma, as well as drug abuse during puberty (Brown, 2011; Meyer, 2019). Intricate interactions between several genetic and/or environmental risk factors may be necessary to affect early neurodevelopmental processes and change the neurodevelopmental trajectories towards the lasting brain disturbances that characterise SZ (Insel, 2010) and to cause the varying psychiatric symptoms and clinical course of the disorder (Daskalakis and Binder, 2015). However, the precise nature of these interactions and their neuropathological consequences remain ill-defined and await future examination.

Besides dysfunctional neuronal signalling, the pathophysiology of SZ appears to involve molecular and functional abnormalities in glial cells, including microglia and macroglia, the latter of which comprises astrocytes, oligodendrocytes and glial progenitor cells (GPCs) (Dietz et al., 2020). This article provides a concise overview of the current evidence supporting altered astrocyte activity in SZ, which ranges from findings obtained from post-mortem immunohistochemical analyses, genetic association studies and transcriptomic investigations, as well as from experimental investigations of astrocyte functions in animal models. Integrating the existing data from these research areas suggests that astrocytes have the capacity to critically affect key neurodevelopmental and homeostatic processes pertaining to SZ pathogenesis, including glutamatergic signalling, synaptogenesis, synaptic pruning and myelination. The further elucidation of astrocyte functions in health and disease may, therefore, offer new insights into how these glial cells contribute to abnormal brain development and functioning underling this debilitating mental disorder.

## Astrocytes – a brief overview

Like neurons and oligodendrocytes, astrocytes originate from neuroepithelium-derived radial glial cells (Kriegstein and Alvarez-Buylla, 2009). Studies in rodents have shown that embryonic astrogliogenesis commences at gestation day (GD) 16–18 in the developing cortex, giving rise to a first fraction of astrocytes (Reemst et al., 2016; Verkhratsky and Nedergaard, 2018). The majority of astrocytes, however, develop during the second wave of astrogliogenesis, which in rodents occurs during postnatal weeks 2 and 3 (Bosworth and Allen, 2017; Verkhratsky and Nedergaard, 2018). As discussed in more detail below, this second wave of astrogliogenesis coincides with the postnatal phases of increased synaptogenesis, synaptic maturation, synaptic pruning and myelination (Bosworth and Allen, 2017; Downes and Mullins, 2014).

In the adult mammalian brain, astrocytes are highly heterogenous (Verkhratsky and Nedergaard, 2018). Once believed to serve as mere supporting cells for neurons, the functional roles of astrocytes are now known to be highly diverse. Astrocytes express a wide range of receptors, transporters, enzymes and ion channels, through which they control and enable homeostasis of ions, pH, neurotransmitters, reactive oxygen species and nutrients (for a comprehensive review, see Verkhratsky and Nedergaard, 2018). They are in close structural association with synapses, with estimations suggesting that one protoplasmic astrocyte contacts and integrates 20–120,000 synapses in mice and up to 2 million synapses in humans (Verkhratsky and Nedergaard, 2018). This special feature of astrocytes has led to the proposal of the tripartite synapse model, which highlights the contribution of astrocytes in regulating synaptic transmission (Araque et al., 1999; Perea and Araque, 2010; Perea et al., 2009). According to this model, astrocytes actively participate in synaptic transmissions by means of neurotransmitter synthesis, buffering and recycling, as well as the secretion of neuromodulators (Mahmoud et al., 2019; Verkhratsky and Nedergaard, 2018).

## Current evidence for astrocytic anomalies in SZ

Evidence for altered astrocyte activity in SZ is manifold and includes findings from post-mortem immunohistochemical analyses, genetic association studies and transcriptomic investigations. Although substantial heterogeneity exists between studies, several post-mortem immunohistochemical findings in SZ identified significant changes in astrocytic density and/or morphology, along with deregulated expression of astrocyte-defining cellular markers (Kim et al., 2018; Tarasov et al., 2019; Trépanier et al., 2016; Zhang et al., 2020). A number of studies reported decreased astrocyte densities in cingulate cortex, motor cortex, medial and ventrolateral regions of the nucleus accumbens, basal nuclei and substantia nigra, whereas the number of astrocytes appear unaltered in the temporal and frontal cortex, amygdala, hippocampus and ventral pallidum (Tarasov et al., 2019). A meta-analysis of glial fibrillary acidic protein (GFAP), a type III intermediate filament protein that is expressed in astrocytes, suggests that there is marked heterogeneity between individual studies, with 6 studies reporting a significant increase in the levels of GFAP in SZ relative to controls, 6 identifying a significant decrease in SZ relative to controls and 21 detecting no significant changes (Trépanier et al., 2016). Similar heterogeneity was also found in post-mortem studies using other astrocytic markers, including aldehyde dehydrogenase 1 (ALDH1) and the calcium binding protein S100β (Steiner et al., 2008; Tarasov et al., 2019; Trépanier et al., 2016; Zhang et al., 2020). As discussed in detail elsewhere (Catts et al., 2014; Kim et al., 2018; Purves-Tyson et al., 2021; Steiner et al., 2008; Tarasov et al., 2019; Trépanier et al., 2016), a number of methodological and patient-related factors, including differences in the brain regions analysed, selection of astrocyte-defining cellular markers, disease state of study subjects and other confounding factors such as medication, smoking and/or suicide, likely contribute to the notable variability in post-mortem findings of altered astrocytic density and/or morphology in SZ.

Genetic association studies and transcriptomic investigations provide additional lines of evidence for an involvement of astrocytic anomalies in SZ. For example, a functional gene set analysis of genome-wide association data from 13,689 individuals with SZ and 18,226 healthy controls found 6 astrocyte gene sets and 3 oligodendrocyte gene sets to be strongly associated with an increased risk for SZ (Goudriaan et al., 2014). Interestingly, the same study found no association of SZ with the microglia-defining gene sets, consistent with findings from the RNA sequencing (RNAseq) analysis from the PsychENCODE Consortium, which integrated genetic and genomic data from ~2000 well-curated, high-quality post-mortem brain samples from individuals with SZ, bipolar disorder (BD), autism spectrum disorder (ASD) and control subjects (Wang et al., 2018). In a transcriptome-wide association study performed by the PsychENCODE Consortium, it was found that astrocyte and interferon (IFN)-response gene expression modules were up-regulated in SZ and ASD, while at the same time, the microglia gene expression module was upregulated only in ASD but down-regulated in SZ and BD (Gandal et al., 2018). Moreover, in a re-analysis of 15 publicly available expression data obtained from bulk tissue, it was found that the expression profiles of cortical astrocytes were consistently increased in both SZ and BD, while the ones from fast-spiking parvalbumin (PV) interneurons were consistently decreased (Toker et al., 2018). In line with these findings, Ramaker et al. showed an increase in astrocytic gene expression and a concomitant decrease in neuron-specific gene expression in the cingulate cortex of SZ and BD patients relative to controls (Ramaker et al., 2017). Finally, a recent RNAseq-based gene set enrichment study found that rare genetic variants of SZ were enriched in astrocytic gene co-expression modules (González-Peñas et al., 2019). Taken together, there is accumulating evidence for a genetic basis of astrocytic anomalies in SZ, which tentatively point towards an upregulation of astrocytic gene expression mostly in cortical areas of the brain.

Experimental studies in rodent models further corroborate a link between astrocytes and SZ. For example, transgenic mice that express a mutant form of the disrupted in schizophrenia 1 (DISC1) gene specifically in astrocytes display behavioural phenotypes relevant for SZ (Ma et al., 2013; Terrillion et al., 2017). DISC1 is considered a ‘historical’ candidate gene for SZ, whose relevance has been challenged by more recent unbiased genome-wide association studies (Farrell et al., 2015; Mathieson et al., 2012). Accumulating evidence now suggests that DISC is generally involved in brain development and functioning through acting on numerous cellular processes, and as such, abnormal DISC1 expression may be a general risk factor for multiple psychiatric disorders rather than constituting a ‘SZ risk gene’ (Facal and Costas, 2019; Niwa et al., 2016; Ryan et al., 2018). Furthermore, astrocytic glutamate/aspartate transporter (GLAST or excitatory amino-acid transporter 1 (EAAT1)) mutant mice were shown to exhibit abnormalities on behavioural measures thought to model the positive, negative and cognitive symptoms of SZ, some of which were rescued by treatment with antipsychotic drugs (Karlsson et al., 2008, 2009; Matsugami et al., 2006). In another study, Windrem et al. developed a chimeric mouse model, in which human GPCs from individuals with SZ or age-matched healthy controls were engrafted into neonatal mice. Mice with GPCs obtained from SZ showed significant changes in astrocyte differentiation and morphology and developed behavioural and brain morphological changes relevant to SZ (Windrem et al., 2017).

## Functional role of astrocytes in synaptogenesis and synapse elimination

Aberrant synaptic formation and elimination have emerged as important pathophysiological processes contributing to SZ and related neurodevelopmental disorders (Boksa, 2012; Keshavan et al., 2020; Sakai, 2020; Sellgren et al., 2019). Indeed, given that SZ is associated with reduced prefrontal grey matter volume (Zhang et al., 2016), decreased synaptic density (Berdenis Van Berlekom et al., 2020), and hypoconnectivity in different brain networks (Li et al., 2019), it has been suggested that SZ could primarily represent a disorder of reduced synapse formation and/or excessive elimination.

In attempts to identify the cellular processes underlying abnormal synaptic refinement in SZ, increasing attention is being paid on microglia. Microglia are innate immune cells of mesodermal origin residing in the brain parenchyma and are pivotal for various immune responses in the brain (Mattei and Notter, 2020). Besides their classical immunological functions, they are actively involved in the refinement of brain circuitries by mediating the elimination of superfluous synapses through complement-dependent phagocytosis (Hong et al., 2016; Paolicelli et al., 2011; Stevens et al., 2007). The association between allelic variations in the complement component 4 (C4) gene – shown to be involved in microglia-dependent synaptic pruning – and the risk to develop SZ strongly supports the hypothesis that excessive synaptic elimination is involved in the pathophysiology of SZ (Sekar et al., 2016).

In addition to microglia, however, emerging evidence points towards a critical involvement of astrocytes in both synaptogenesis (Bosworth and Allen, 2017; Verkhratsky and Nedergaard, 2018) and synaptic elimination (Bialas and Stevens, 2013; Chung et al., 2013; Iino et al., 2001; Vainchtein et al., 2018; Yang et al., 2016) during postnatal brain maturation. Astrocytes have been shown to produce and secrete numerous synaptogenic factors, which regulate synapse formation in a highly complex, brain region- and neuronal subtype-specific manner (as reviewed by Bosworth and Allen, 2017). Among these is the fatty acid binding protein 7 (FABP7), which in turn has been implicated in SZ (Shimamoto et al., 2014; Watanabe et al., 2007). In mice, FABP7 has been identified as a critical synaptogenic factor, especially for synaptogenesis occurring in the medial prefrontal cortex (mPFC) (Ebrahimi et al., 2016). Animals that do not express FABP7 display decreased prefrontal dendritic complexity, spine density and maturity of spines and show behavioural deficits relevant for SZ and related disorders (Ebrahimi et al., 2016; Shimamoto et al., 2014).

Thus far, astrocyte-dependent synaptic elimination has been described in the developmental refinement of retinogeniculate connectivity in the visual system (Bialas and Stevens, 2013; Chung et al., 2013; Vainchtein et al., 2018), the ventral trigeminothalamic tract of the somatosensory system (Yang et al., 2016) and the cerebellar connectivity between climbing fibres and Purkinje cells (Iino et al., 2001). There are several known mechanisms by which astrocytes eliminate synapses. First, they can eliminate synapses via phagocytosis, which appears to involve astrocytic activation of phagocytosis-stimulating receptors, including mer receptor tyrosine kinase (MERTK) and multiple epidermal growth factor-like domain protein 10 (MEGF10) (Chung et al., 2013). Second, astrocytes produce and release the cytokines interleukin-33 (IL-33) (Vainchtein et al., 2018) and transforming growth factor-β (TGF-β) (Bialas and Stevens, 2013), which in turn stimulate microglia to phagocytose synapses in a complement-dependent manner. A third mechanism by which astrocytes promote synaptic elimination involves activation of the intracellular inositol-1,4,5-triphosphate (IP3) pathway and subsequent release of Ca^2+^ from endoplasmic reticulum (Yang et al., 2016). While the precise cellular mechanisms remain to be identified, these data indicate that astrocytes can promote synaptic elimination in a Ca^2+^-dependent manner.

As it has been proposed for microglia (Sekar et al., 2016; Sellgren et al., 2019), alterations in astrocyte activity during critical developmental time windows could thus mediate excessive elimination of synapses, resulting in functional dysconnectivity of different brain regions and disturbances in distinct neurotransmitter systems (Insel, 2010) and ultimately increase the risk of SZ and related disorders.

## Functional role of astrocytes in white matter integrity and myelination

Impaired white matter integrity is another hallmark pathology implicated in SZ (Dietz et al., 2020; Kelly et al., 2018; Landek-Salgado et al., 2016). White matter comprises long-ranging axonal connections that are ensheathed and insulated by myelin – a lipid-dense material produced by oligodendrocytes assuring fast action potential propagation. White matter integrity can be assessed in-vivo using diffusion tensor imaging (DTI) with magnetic resonance imaging (MRI) and indexed by changes in fractional anisotropy (FA), a measure of diffusion rate along an axon (Chang et al., 2017). Several studies found reduced FA in SZ compared to healthy controls, especially within white matter areas of the forebrain (Kanaan et al., 2009; Kelly et al., 2018; Samartzis et al., 2014), indicating impaired white matter integrity in SZ relative to controls. Interestingly, such changes in white matter could already be detected in high-risk individuals before the presence of obvious clinical symptoms, and these abnormalities appear to progress along the clinical course of SZ (Samartzis et al., 2014). In support of the findings provided by imaging studies, post-mortem investigations have also provided immunohistochemical and/or transcriptomic evidence for myelination deficits in SZ (Landek-Salgado et al., 2016).

Although myelin sheaths that insulate axons are produced by oligodendrocytes, astrocytes have been shown to critically influence this process (Molina-Gonzalez and Miron, 2019). For example, astrocytes directly provide oligodendrocytes with nutrients and substrates via gap junctions, which are indispensable for the metabolically highly demanding process of myelin formation and maintenance (Orthmann-Murphy et al., 2008). Furthermore, disrupting the direct communication between astrocytes and oligodendrocytes results in severe myelin disorders, which have been associated with SZ-like psychosis (Molina-Gonzalez and Miron, 2019; Walterfang et al., 2005). Hence, astrocytes are key for maintaining white matter integrity and myelination, such that disrupting astrocytic activity may lead to impaired white matter structure and function pertaining to SZ and beyond.

This hypothesis has recently been supported by an elegant experimental study, where neonatal myelin-deficient mice (Windrem et al., 2017) were engrafted with human GPCs produced from induced pluripotent stem cells (iPSCs) of individuals with SZ and age-matched controls (Windrem et al., 2017). GPCs are a proliferating cell type of the central nervous system (CNS) that have the capacity to differentiate into oligodendrocytes or astrocytes, following a strictly regulated pathway (Dietz et al., 2020; Hill and Nishiyama, 2014). In the study by Windrem et al. (2017), it was found that GPCs generated from individuals with SZ migrated prematurely into the cortex, which led to decreased expansion of the white matter and hypomyelination relative to control GPCs (Windrem et al., 2017). The authors further discovered that chimeras with GPCs from individuals with SZ showed delayed astrocyte differentiation and developed abnormal behaviours, including impaired sensorimotor gating, increased anxiety-like behaviour and reduced social approach behaviour (Windrem et al., 2017). Finally, RNAseq of cultured schizophrenic GPCs revealed alterations in the expression of genes associated with glial differentiation, as well as synaptic transmission, development and function, indicating that the observed glial pathology was cell autonomous (Windrem et al., 2017).

## Functional role of astrocyte in glutamatergic neurotransmission

The functional role of astrocytes in regulating glutamatergic neurotransmission has long been known (Hansson and Rönnbäck, 1995; Mahmoud et al., 2019). More recently, it has been proposed that astrocytes could contribute to some of the alterations in the glutamatergic neurotransmitter system associated with SZ (Mei et al., 2018; Tarasov et al., 2019). The glutamate hypothesis of SZ was initially postulated based on the psychotogenic effects of N-methyl-d-aspartate receptor (NMDAR) antagonists, such as phencyclidine (PCP) and ketamine (Javitt and Zukin, 1991; Luby et al., 1962; Moghaddam and Javitt, 2012). Subsequently, this hypothesis received support from several research lines, including post-mortem studies revealing lower mRNA and protein expression of specific NMDAR subunits and changes in the postsynaptic density of glutamatergic synapses in the brains of individuals with SZ (Balu, 2016; Banerjee et al., 2015). Taken together, the glutamate hypothesis of SZ suggests that (at least parts of) SZ-associated symptoms underly NMDAR hypofunctions in discrete brain regions, including the prefrontal cortex (PFC) and hippocampus (Moghaddam and Javitt, 2012), and in specific cell types. With regards to the latter, hypofunction of NMDA receptors on PV-positive interneurons appears particularly relevant to the pathophysiology of SZ, resulting in decreased activity in PV-positive GABAergic interneurons causing an imbalance in the excitatory/inhibitory neurotransmitter actions (Gonzalez-Burgos and Lewis, 2012). This view is in line with findings in mice where the NR1 subunit of the NMDAR has been ablated specifically in PV-positive GABAergic neurons, which in turn led to the development of neuromorphological and behavioural changes reminiscent of SZ (Belforte et al., 2010). A role of aberrant NMDAR signalling in SZ has been further corroborated by genome-wide association studies identifying genes encoding NMDAR subunits to be genetic risk factors for SZ (Allen et al., 2008; Consortium, 2014). Furthermore, single nucleotide polymorphisms of genes involved in the synthesis of D-serine, a co-activator of NMDAR, have been associated with SZ (Boks et al., 2007).

### Glutamate

Despite substantial heterogeneity, numerous studies using proton magnetic resonance spectroscopy (^1^H-MRS) have provided evidence for altered glutamate levels in unmedicated individuals with SZ (Poels et al., 2014). While initial findings suggested that individuals with SZ display increased glutamate levels in the PFC, presumably as a result of increased presynaptic release (Mei et al., 2018; Poels et al., 2014), more recent ^1^H-MRS studies found lower levels of glutamate in prefrontal brain regions of individuals with SZ relative to healthy controls (Reid et al., 2019; Wang et al., 2019). The precise source of these discrepant findings remains elusive but may involve age differences in the study sample (Brandt et al., 2016) and/or technical differences in the capacity of ^1^H-MRS to separate glutamate and glutamine into distinct signals (Reid et al., 2019; Wang et al., 2019). Both ends of the spectrums (i.e. reduced or increased glutamate levels) may, however, involve altered astrocytic mechanisms. Indeed, astrocytes replenish presynaptic vesicles with synaptically released glutamate via the glutamate-glutamine cycle, a process shown to be disturbed in individuals with SZ (Dietz et al., 2020; Hertz and Rothman, 2016; Jelen et al., 2018; Mei et al., 2018). In this cycle, astrocytes buffer glutamate from the synaptic cleft via glutamate transporters, including EAATs, and then convert glutamate into glutamine via the glutamine synthetase (Hertz and Rothman, 2016; Mahmoud et al., 2019). Upon its release by astrocytes, glutamine is taken up by neurons, converted back into glutamate, packed into presynaptic vesicles, and stored in synaptic terminals (Dietz et al., 2020; Hertz and Rothman, 2016). In addition to their role in the glutamate-glutamine cycle, astrocytes also represent the primary cell type that synthesizes glutamate from glucose, thus providing another astrocyte-mediated process that regulates presynaptic glutamate levels in the brain (Mei et al., 2018). Disruptions of astrocyte functions pertaining to the glutamate-glutamine cycle and/or de-novo synthesis of glutamate from glucose can thus be expected to contribute to abnormal glutamate levels as seen in SZ and related disorders.

Moreover, deficient or excessive glutamate clearance from the synaptic cleft by astrocytes could yet provide another cellular mechanism for abnormal glutamate signalling in SZ. In the adult brain, EAATs have been shown to play a major role in glutamate uptake from the synaptic cleft (Bar-Peled et al., 1997; Haugeto et al., 1996; Hu et al., 2015; Robinson, 1998). Among the four subtypes of EAATs, EAAT1 and EAAT2 have been shown to be predominantly expressed in astrocytes (Haugeto et al., 1996). In the adult brain, EAAT2 has been identified to be responsible for approximately 90% of total glutamate uptake in the synaptic cleft (Bar-Peled et al., 1997; Robinson, 1998). Changes in EAATs expression and function could therefore directly alter synaptic glutamate levels and glutamatergic neurotransmission, thereby precipitating the glutamatergic pathophysiology of SZ and related disorders. In support of this notion, post-mortem studies found abnormal mRNA and protein expression levels of EAAT1 and EAAT2 in different brain regions of SZ patients relative to controls (Hu et al., 2015; Katsel et al., 2011; McCullumsmith et al., 2016; Mei et al., 2018; O’Donovan et al., 2017). A link between abnormal EAAT expression and SZ-related pathology was also established by work in animal models, which demonstrated that genetically induced loss of astrocytic EAATs causes behavioural phenotypes implicated in SZ and related psychotic disorders (Karlsson et al., 2008, 2009; Matsugami et al., 2006).

### D-serine

NMDAR activation necessitates the binding of both glutamate and a co-agonist, with D-serine and glycine representing the endogenous co-activators (Hu et al., 2015). In support of the glutamate hypothesis of SZ, several lines of evidence indicate that deficits in the availability of D-serine may contribute to NMDAR hypofunctions associated with SZ (Hu et al., 2015; Labrie et al., 2012; Mei et al., 2018). D-serine, on one hand, is catalysed from L-serine by the enzyme serine racemase (SR), which is primarily expressed in neurons (Balu et al., 2014; Kartvelishvily et al., 2006; Miya et al., 2008). L-serine, on the other hand, is de novo synthesed in astrocytes involving the enzyme 3-phosphoglycerate dehydrogenase (PHGDH) (Yamasaki et al., 2001). Deletion of astrocytic PHGDH reduced L-serine levels, as well as neuronal D-serine (Neame et al., 2019; Yang et al., 2010). Moreover, selective inhibition of PHGDH reduced L- and D-serine synthesis and reduced the NMDAR synaptic potentials and long-term potentiation (LTP) at the Schaffer collaterals-CA1 synapses in the hippocampus (Neame et al., 2019). Further experimental work in animal models highlight that there may be a critical link between abnormal astrocyte functions, D-serine productions and SZ-related pathologies. Based on the accumulating evidence implicating the DISC1 gene in the aetiology of mental illnesses (Chubb et al., 2008; Facal and Costas, 2019; Ishizuka et al., 2006; Niwa et al., 2016; Ryan et al., 2018; Sawa and Snyder, 2005), Ma et al. (2013) generated mice expressing a mutant form of DISC1 selectively in astrocytes. Compared with wild-type controls, these mice displayed decreased levels of D-serine and a number of behavioural abnormalities relevant to SZ and other mental illnesses, including locomotor hyperactivity, deficits in sensorimotor gating in the form of prepulse inhibition, increased anxiety-like behaviour, impairments in social interaction and recognition, as well as cognitive impairments (Ma et al., 2013; Terrillion et al., 2017). Intriguingly, chronic treatment with D-serine significantly improved the behavioural phenotypes in mutant DISC1 mice (Ma et al., 2013; Terrillion et al., 2017). A more recent study showed that region-specific knockdown (KD) of DISC-1 in mature mouse astrocytes affected cognitive performance in a brain region-specific manner (Shevelkin et al., 2020). Subsequent post-mortem analyses in this model revealed that astrocytes-specific DISC1-KD caused marked alterations in the morphology of astrocytes, the expression of mitochondrial markers and astrocytic EAAT1, as well as the levels of glutamatergic and GABAergic synaptic markers (Shevelkin et al., 2020). The translational value of these findings is supported by post-mortem findings showing reduced density of DISC1-expressing astrocytes in the hippocampus of SZ patients relative to healthy controls (Bernstein et al., 2018).

## Concluding remarks

Concurrent with dysfunctional neuronal signalling, the pathophysiology of SZ involves molecular and functional abnormalities in glial cells, including astrocytes. The current evidence supporting altered astrocyte activity in SZ ranges from findings obtained from post-mortem analyses, genetic association studies and transcriptomic investigations, as well as from experimental investigations of astrocyte functions in animal models. Integrating the existing data from these research areas strongly suggests that astrocytes have the capacity to critically affect key neurodevelopmental and homeostatic processes pertaining to SZ pathogenesis, including glutamatergic signalling, synaptogenesis, synaptic pruning and myelination. The further elucidation of astrocytes functions in health and disease may, therefore, offer new insights into how these glial cells contribute to abnormal brain development and functioning underlying this debilitating mental disorder.

## References

[bibr1-23982128211009148] Addington J Heinssen R (2012) Prediction and prevention of psychosis in youth at clinical high risk. Annual Review of Clinical Psychology 8: 269–289. 10.1146/annurev-clinpsy-032511-143146 22224837

[bibr2-23982128211009148] Allen NC Bagade S McQueen MB, et al. (2008) Systematic meta-analyses and field synopsis of genetic association studies in schizophrenia: The SzGene database. Nature Genetics 40(7): 827–834. 18583979 10.1038/ng.171

[bibr3-23982128211009148] Araque A Sanzgiri RP Parpura V, et al. (1999) Astrocyte-induced modulation of synaptic transmission. Canadian Journal of Physiology and Pharmacology 77(9): 699–706. 10566947

[bibr4-23982128211009148] Balu DT (2016) The NMDA receptor and schizophrenia: From pathophysiology to treatment. Advances in Pharmacology 76: 351–382. 27288082 10.1016/bs.apha.2016.01.006 PMC5518924

[bibr5-23982128211009148] Balu DT Takagi S Puhl MD, et al. (2014) D-serine and serine racemase are localized to neurons in the adult mouse and human forebrain. Cellular and Molecular Neurobiology 34(3): 419–435. 24436034 10.1007/s10571-014-0027-z PMC4505359

[bibr6-23982128211009148] Banerjee A Wang HY Borgmann-Winter KE, et al. (2015) Src kinase as a mediator of convergent molecular abnormalities leading to NMDAR hypoactivity in schizophrenia. Molecular Psychiatry 20(9): 1091–1100. 25330739 10.1038/mp.2014.115 PMC5156326

[bibr7-23982128211009148] Bar-Peled O Ben-Hur H Biegon A, et al. (1997) Distribution of glutamate transporter subtypes during human brain development. Journal of Neurochemistry 69(6): 2571–2580. 9375691 10.1046/j.1471-4159.1997.69062571.x

[bibr8-23982128211009148] Belforte JE Zsiros V Sklar ER, et al. (2010) Postnatal NMDA receptor ablation in corticolimbic interneurons confers schizophrenia-like phenotypes. Nature Neuroscience 13(1): 76–83. 19915563 10.1038/nn.2447 PMC2797836

[bibr9-23982128211009148] Berdenis Van Berlekom A Muflihah CH Snijders GJLJ, et al. (2020) Synapse pathology in schizophrenia: A meta-analysis of postsynaptic elements in postmortem brain studies. Schizophrenia Bulletin 46(2): 374–386. 31192350 10.1093/schbul/sbz060 PMC7442385

[bibr10-23982128211009148] Bernstein HG Dobrowolny H Keilhoff G, et al. (2018) Reduced density of DISC1 expressing astrocytes in the dentate gyrus but not in the subventricular zone in schizophrenia. Neuropsychopharmacology 43(3): 457–458. 29326433 10.1038/npp.2017.242 PMC5770772

[bibr11-23982128211009148] Bialas AR Stevens B (2013) TGF-β signaling regulates neuronal C1q expression and developmental synaptic refinement. Nature Neuroscience 16(12): 1773–1782. 24162655 10.1038/nn.3560 PMC3973738

[bibr12-23982128211009148] Boks MP Rietkerk T van de Beek MH, et al. (2007) Reviewing the role of the genes G72 and DAAO in glutamate neurotransmission in schizophrenia. European Neuropsychopharmacology 17(9): 567–572. 17250995 10.1016/j.euroneuro.2006.12.003

[bibr13-23982128211009148] Boksa P (2012) Abnormal synaptic pruning in schizophrenia: Urban myth or reality? Journal of Psychiatry & Neuroscience 37(2): 75–77. 22339991 10.1503/jpn.120007 PMC3297065

[bibr14-23982128211009148] Bosworth AP Allen NJ (2017) The diverse actions of astrocytes during synaptic development. Current Opinion in Neurobiology 47: 38–43. 28938161 10.1016/j.conb.2017.08.017

[bibr15-23982128211009148] Brandt AS Unschuld PG Pradhan S, et al. (2016) Age-related changes in anterior cingulate cortex glutamate in schizophrenia: A (1)H MRS Study at 7 Tesla. Schizophrenia Research 172(1–3): 101–105. 26925800 10.1016/j.schres.2016.02.017 PMC4821673

[bibr16-23982128211009148] Brown AS (2011) The environment and susceptibility to schizophrenia. Progress in Neurobiology 93(1): 23–58. 20955757 10.1016/j.pneurobio.2010.09.003 PMC3521525

[bibr17-23982128211009148] Catts VS Wong J Fillman SG, et al. (2014) Increased expression of astrocyte markers in schizophrenia: Association with neuroinflammation. Australian and New Zealand Journal of Psychiatry 48(8): 722–734. 10.1177/0004867414531078 24744400

[bibr18-23982128211009148] Chang EH Argyelan M Aggarwal M, et al. (2017) The role of myelination in measures of white matter integrity: Combination of diffusion tensor imaging and two-photon microscopy of CLARITY intact brains. NeuroImage 147: 253–261. 27986605 10.1016/j.neuroimage.2016.11.068 PMC5560594

[bibr19-23982128211009148] Chubb JE Bradshaw NJ Soares DC, et al. (2008) The DISC locus in psychiatric illness. Molecular Psychiatry 13(1): 36–64. 17912248 10.1038/sj.mp.4002106

[bibr20-23982128211009148] Chung WS Clarke LE Wang GX, et al. (2013) Astrocytes mediate synapse elimination through MEGF10 and MERTK pathways. Nature 504(7480): 394–400. 24270812 10.1038/nature12776 PMC3969024

[bibr21-23982128211009148] Consortium SWGotPG (2014) Biological insights from 108 schizophrenia-associated genetic loci. Nature 511(7510): 421–427. 25056061 10.1038/nature13595 PMC4112379

[bibr22-23982128211009148] Correll CU Schooler NR (2020) Negative symptoms in schizophrenia: A review and clinical guide for recognition, assessment, and treatment. Neuropsychiatric Disease and Treatment 16: 519–534. 32110026 10.2147/NDT.S225643 PMC7041437

[bibr23-23982128211009148] Daskalakis NP Binder EB (2015) Schizophrenia in the spectrum of gene-stress interactions: The FKBP5 example. Schizophrenia Bulletin 41(2): 323–329. 25592294 10.1093/schbul/sbu189 PMC4332957

[bibr24-23982128211009148] Dietz AG Goldman SA Nedergaard M (2020) Glial cells in schizophrenia: A unified hypothesis. The Lancet Psychiatry 7(3): 272–281. 31704113 10.1016/S2215-0366(19)30302-5 PMC7267935

[bibr25-23982128211009148] Downes N Mullins P (2014) The development of myelin in the brain of the juvenile rat. Toxicologic Pathology 42(5): 913–922. 24129760 10.1177/0192623313503518

[bibr26-23982128211009148] Ebrahimi M Yamamoto Y Sharifi K, et al. (2016) Astrocyte-expressed FABP7 regulates dendritic morphology and excitatory synaptic function of cortical neurons. Glia 64(1): 48–62. 26296243 10.1002/glia.22902

[bibr27-23982128211009148] Facal F Costas J (2019) Evidence of association of the DISC1 interactome gene set with schizophrenia from GWAS. Progress in Neuro-Psychopharmacology & Biological Psychiatry 95: 109729. 10.1016/j.pnpbp.2019.109729 31398428

[bibr28-23982128211009148] Farrell MS Werge T Sklar P, et al. (2015) Evaluating historical candidate genes for schizophrenia. Molecular Psychiatry 20(5): 555–562. 25754081 10.1038/mp.2015.16 PMC4414705

[bibr29-23982128211009148] Gandal MJ Zhang P Hadjimichael E, et al. (2018) Transcriptome-wide isoform-level dysregulation in ASD, schizophrenia, and bipolar disorder. Science 362(6420): eaat8127. 10.1126/science.aat8127 PMC6443102 30545856

[bibr30-23982128211009148] Gonzalez-Burgos G Lewis DA (2012) NMDA receptor hypofunction, parvalbumin-positive neurons, and cortical gamma oscillations in schizophrenia. Schizophrenia Bulletin 38(5): 950–957. 22355184 10.1093/schbul/sbs010 PMC3446219

[bibr31-23982128211009148] González-Peñas J Costas J Villamayor MJG, et al. (2019) Enrichment of rare genetic variants in astrocyte gene enriched co-expression modules altered in postmortem brain samples of schizophrenia. Neurobiology of Disease 121: 305–314. 30347265 10.1016/j.nbd.2018.10.013

[bibr32-23982128211009148] Goudriaan A De Leeuw C Ripke S, et al. (2014) Specific glial functions contribute to schizophrenia susceptibility. Schizophrenia Bulletin 40(4): 925–935. 23956119 10.1093/schbul/sbt109 PMC4059439

[bibr33-23982128211009148] Hansson E Rönnbäck L (1995) Astrocytes in glutamate neurotransmission. The FASEB Journal 9(5): 343–350. 7534736 10.1096/fasebj.9.5.7534736

[bibr34-23982128211009148] Haugeto O Ullensvang K Levy LM, et al. (1996) Brain glutamate transporter proteins form homomultimers. Journal of Biological Chemistry 271(44): 27715–27722. 10.1074/jbc.271.44.27715 8910364

[bibr35-23982128211009148] Hertz L Rothman DL (2016) Glucose, lactate, β-hydroxybutyrate, acetate, GABA, and succinate as substrates for synthesis of glutamate and GABA in the glutamine-glutamate/GABA cycle. Advances in Neurobiology 13: 9–42. 27885625 10.1007/978-3-319-45096-4_2

[bibr36-23982128211009148] Hill RA Nishiyama A (2014) NG2 cells (polydendrocytes): Listeners to the neural network with diverse properties. Glia 62(8): 1195–1210. 24753030 10.1002/glia.22664 PMC4282324

[bibr37-23982128211009148] Hong S Dissing-Olesen L Stevens B (2016) New insights on the role of microglia in synaptic pruning in health and disease. Current Opinion in Neurobiology 36: 128–134. 26745839 10.1016/j.conb.2015.12.004 PMC5479435

[bibr38-23982128211009148] Hu W MacDonald ML Elswick DE, et al. (2015) The glutamate hypothesis of schizophrenia: Evidence from human brain tissue studies. Annals of the New York Academy of Sciences 1338(1): 38–57. 25315318 10.1111/nyas.12547 PMC4363164

[bibr39-23982128211009148] Iino M Goto K Kakegawa W, et al. (2001) Glia-synapse interaction through Ca^2+^-permeable AMPA receptors in Bergmann glia. Science 292(5518): 926–929. 11340205 10.1126/science.1058827

[bibr40-23982128211009148] Insel TR (2010) Rethinking schizophrenia. Nature 468(7321): 187–193. 21068826 10.1038/nature09552

[bibr41-23982128211009148] Ishizuka K Paek M Kamiya A, et al. (2006) A review of Disrupted-In-Schizophrenia-1 (DISC1): Neurodevelopment, cognition, and mental conditions. Biological Psychiatry 59(12): 1189–1197. 16797264 10.1016/j.biopsych.2006.03.065

[bibr42-23982128211009148] Javitt DC Zukin SR (1991) Recent advances in the phencyclidine model of schizophrenia. American Journal of Psychiatry 148(10): 1301–1308. 10.1176/ajp.148.10.1301 1654746

[bibr43-23982128211009148] Jelen LA King S Mullins PG, et al. (2018) Beyond static measures: A review of functional magnetic resonance spectroscopy and its potential to investigate dynamic glutamatergic abnormalities in schizophrenia. Journal of Psychopharmacology 32(5): 497–508. 29368979 10.1177/0269881117747579

[bibr44-23982128211009148] Kanaan R Barker G Brammer M, et al. (2009) White matter microstructure in schizophrenia: Effects of disorder, duration and medication. British Journal of Psychiatry 194(3): 236–242. 10.1192/bjp.bp.108.054320 PMC2802507 19252154

[bibr45-23982128211009148] Karlsson RM Tanaka K Heilig M, et al. (2008) Loss of glial glutamate and aspartate transporter (excitatory amino acid transporter 1) causes locomotor hyperactivity and exaggerated responses to psychotomimetics: Rescue by haloperidol and metabotropic glutamate 2/3 agonist. Biological Psychiatry 64(9): 810–814. 18550032 10.1016/j.biopsych.2008.05.001 PMC2696047

[bibr46-23982128211009148] Karlsson RM Tanaka K Saksida LM, et al. (2009) Assessment of glutamate transporter GLAST (EAAT1)-deficient mice for phenotypes relevant to the negative and executive/cognitive symptoms of schizophrenia. Neuropsychopharmacology 34(6): 1578–1589. 19078949 10.1038/npp.2008.215 PMC3400972

[bibr47-23982128211009148] Kartvelishvily E Shleper M Balan L, et al. (2006) Neuron-derived D-serine release provides a novel means to activate N-methyl-D-aspartate receptors. Journal of Biological Chemistry 281(20): 14151–14162. 10.1074/jbc.M512927200 16551623

[bibr48-23982128211009148] Katsel P Byne W Roussos P, et al. (2011) Astrocyte and glutamate markers in the superficial, deep, and white matter layers of the anterior cingulate gyrus in schizophrenia. Neuropsychopharmacology 36(6): 1171–1177. 21270770 10.1038/npp.2010.252 PMC3077461

[bibr49-23982128211009148] Kelly S Jahanshad N Zalesky A, et al. (2018) Widespread white matter microstructural differences in schizophrenia across 4322 individuals: Results from the ENIGMA Schizophrenia DTI Working Group. Molecular Psychiatry 23(5): 1261–1269. 29038599 10.1038/mp.2017.170 PMC5984078

[bibr50-23982128211009148] Keshavan M Lizano P Prasad K (2020) The synaptic pruning hypothesis of schizophrenia: Promises and challenges. World Psychiatry 19(1): 110–111. 31922664 10.1002/wps.20725 PMC6953570

[bibr51-23982128211009148] Kim R Healey KL Sepulveda-Orengo MT, et al. (2018) Astroglial correlates of neuropsychiatric disease: From astrocytopathy to astrogliosis. Progress in Neuro-Psychopharmacology & Biological Psychiatry 87(Pt A): 126–146. 28989099 10.1016/j.pnpbp.2017.10.002 PMC5889368

[bibr52-23982128211009148] Kriegstein A Alvarez-Buylla A (2009) The glial nature of embryonic and adult neural stem cells. Annual Review of Neuroscience 32: 149–184. 10.1146/annurev.neuro.051508.135600 PMC3086722 19555289

[bibr53-23982128211009148] Labrie V Wong AH Roder JC (2012) Contributions of the D-serine pathway to schizophrenia. Neuropharmacology 62(3): 1484–1503. 21295046 10.1016/j.neuropharm.2011.01.030

[bibr54-23982128211009148] Landek-Salgado MA Faust TE Sawa A (2016) Molecular substrates of schizophrenia: Homeostatic signaling to connectivity. Molecular Psychiatry 21(1): 10–28. 26390828 10.1038/mp.2015.141 PMC4684728

[bibr55-23982128211009148] Lewandowski KE Cohen BM Ongur D (2011) Evolution of neuropsychological dysfunction during the course of schizophrenia and bipolar disorder. Psychological Medicine 41(2): 225–241. 20836900 10.1017/S0033291710001042

[bibr56-23982128211009148] Lewis DA Lieberman JA (2000) Catching up on schizophrenia: Natural history and neurobiology. Neuron 28(2): 325–334. 11144342 10.1016/s0896-6273(00)00111-2

[bibr57-23982128211009148] Li S Hu N Zhang W, et al. (2019) Dysconnectivity of multiple brain networks in schizophrenia: A meta-analysis of resting-state functional connectivity. Frontiers in Psychiatry 10: 482. 31354545 10.3389/fpsyt.2019.00482 PMC6639431

[bibr58-23982128211009148] Lieberman JA Perkins D Belger A, et al. (2001) The early stages of schizophrenia: Speculations on pathogenesis, pathophysiology, and therapeutic approaches. Biological Psychiatry 50(11): 884–897. 11743943 10.1016/s0006-3223(01)01303-8

[bibr59-23982128211009148] Luby ED Gottlieb JS Cohen BD, et al. (1962) Model psychoses and schizophrenia. American Journal of Psychiatry 119: 61–67. 10.1176/ajp.119.1.61 14467063

[bibr60-23982128211009148] Ma TM Abazyan S Abazyan B, et al. (2013) Pathogenic disruption of DISC1-serine racemase binding elicits schizophrenia-like behavior via D-serine depletion. Molecular Psychiatry 18(5): 557–567. 22801410 10.1038/mp.2012.97 PMC3475769

[bibr61-23982128211009148] McCullumsmith RE O’Donovan SM Drummond JB, et al. (2016) Cell-specific abnormalities of glutamate transporters in schizophrenia: Sick astrocytes and compensating relay neurons? Molecular Psychiatry 21(6): 823–830. 26416546 10.1038/mp.2015.148 PMC7584379

[bibr62-23982128211009148] Mahmoud S Gharagozloo M Simard C, et al. (2019) Astrocytes maintain glutamate homeostasis in the CNS by controlling the balance between glutamate uptake and release. Cells 8(2): 184. 10.3390/cells8020184 PMC6406900 30791579

[bibr63-23982128211009148] Mathieson I Munafò MR Flint J (2012) Meta-analysis indicates that common variants at the DISC1 locus are not associated with schizophrenia. Molecular Psychiatry 17(6): 634–641. 21483435 10.1038/mp.2011.41 PMC3359642

[bibr64-23982128211009148] Matsugami TR Tanemura K Mieda M, et al. (2006) From the cover: Indispensability of the glutamate transporters GLAST and GLT1 to brain development. Proceedings of the National Academy of Sciences of the United States of America 103(32): 12161–12166. 16880397 10.1073/pnas.0509144103 PMC1524927

[bibr65-23982128211009148] Mattei D Notter T (2020) Basic concept of microglia biology and neuroinflammation in relation to psychiatry. Current Topics in Behavioral Neurosciences 44: 9–34. 30739307 10.1007/7854_2018_83

[bibr66-23982128211009148] Mei YY Wu DC Zhou N (2018) Astrocytic regulation of glutamate transmission in schizophrenia. Frontiers in Psychiatry 9: 544. 30459650 10.3389/fpsyt.2018.00544 PMC6232167

[bibr67-23982128211009148] Meyer U (2019) Neurodevelopmental resilience and susceptibility to maternal immune activation. Trends in Neurosciences 42(11): 793–806. 31493924 10.1016/j.tins.2019.08.001

[bibr68-23982128211009148] Miya K Inoue R Takata Y, et al. (2008) Serine racemase is predominantly localized in neurons in mouse brain. The Journal of Comparative Neurology 510(6): 641–654. 18698599 10.1002/cne.21822

[bibr69-23982128211009148] Moghaddam B Javitt D (2012) From revolution to evolution: The glutamate hypothesis of schizophrenia and its implication for treatment. Neuropsychopharmacology 37(1): 4–15. 21956446 10.1038/npp.2011.181 PMC3238069

[bibr70-23982128211009148] Molina-Gonzalez I Miron VE (2019) Astrocytes in myelination and remyelination. Neuroscience Letters 713: 134532. 10.1016/j.neulet.2019.134532 31589903

[bibr71-23982128211009148] Neame S Safory H Radzishevsky I, et al. (2019) The NMDA receptor activation by d-serine and glycine is controlled by an astrocytic PHGDH-dependent serine shuttle. Proceedings of the National Academy of Sciences of the United States of America 116(41): 20736–20742. 31548413 10.1073/pnas.1909458116 PMC6789919

[bibr72-23982128211009148] Niwa M Cash-Padgett T Kubo KI, et al. (2016) DISC1 a key molecular lead in psychiatry and neurodevelopment: No-More Disrupted-in-Schizophrenia 1. Molecular Psychiatry 21(11): 1488–1489. 27595595 10.1038/mp.2016.154 PMC5472474

[bibr73-23982128211009148] O’Donovan SM Sullivan CR McCullumsmith RE (2017) The role of glutamate transporters in the pathophysiology of neuropsychiatric disorders. NPJ Schizophrenia 3(1): 32. 28935880 10.1038/s41537-017-0037-1 PMC5608761

[bibr74-23982128211009148] Orthmann-Murphy JL Abrams CK Scherer SS (2008) Gap junctions couple astrocytes and oligodendrocytes. Journal of Molecular Neuroscience 35(1): 101–116. 18236012 10.1007/s12031-007-9027-5 PMC2650399

[bibr75-23982128211009148] Owen MJ Sawa A Mortensen PB (2016) Schizophrenia. The Lancet 388(10039): 86–97. 10.1016/S0140-6736(15)01121-6 PMC4940219 26777917

[bibr76-23982128211009148] Paolicelli RC Bolasco G Pagani F, et al. (2011) Synaptic pruning by microglia is necessary for normal brain development. Science 333(6048): 1456–1458. 21778362 10.1126/science.1202529

[bibr77-23982128211009148] Patel KR Cherian J Gohil K, et al. (2014) Schizophrenia: Overview and treatment options. P & T 39(9): 638–645. 25210417 PMC4159061

[bibr78-23982128211009148] Perea G Araque A (2010) GLIA modulates synaptic transmission. Brain Research Reviews 63(1–2): 93–102. 19896978 10.1016/j.brainresrev.2009.10.005

[bibr79-23982128211009148] Perea G Navarrete M Araque A (2009) Tripartite synapses: Astrocytes process and control synaptic information. Trends in Neurosciences 32(8): 421–431. 19615761 10.1016/j.tins.2009.05.001

[bibr80-23982128211009148] Poels EM Kegeles LS Kantrowitz JT, et al. (2014) Imaging glutamate in schizophrenia: Review of findings and implications for drug discovery. Molecular Psychiatry 19(1): 20–29. 24166406 10.1038/mp.2013.136

[bibr81-23982128211009148] Potkin SG Kane JM Correll CU, et al. (2020) The neurobiology of treatment-resistant schizophrenia: Paths to antipsychotic resistance and a roadmap for future research. NPJ Schizophrenia 6(1): 1. 31911624 10.1038/s41537-019-0090-z PMC6946650

[bibr82-23982128211009148] Purves-Tyson TD Weber-Stadlbauer U Richetto J, et al. (2021) Increased levels of midbrain immune-related transcripts in schizophrenia and in murine offspring after maternal immune activation. Molecular Psychiatry 26(3): 849–863. 31168068 10.1038/s41380-019-0434-0 PMC7910216

[bibr83-23982128211009148] Ramaker RC Bowling KM Lasseigne BN, et al. (2017) Post-mortem molecular profiling of three psychiatric disorders. Genome Medicine 9(1): 72. 28754123 10.1186/s13073-017-0458-5 PMC5534072

[bibr84-23982128211009148] Reemst K Noctor SC Lucassen PJ, et al. (2016) The indispensable roles of microglia and astrocytes during brain development. Frontiers in Human Neuroscience 10: 566. 27877121 10.3389/fnhum.2016.00566 PMC5099170

[bibr85-23982128211009148] Reid MA Salibi N White DM, et al. (2019) 7T proton magnetic resonance spectroscopy of the anterior cingulate cortex in first-episode schizophrenia. Schizophrenia Bulletin 45(1): 180–189. 29385594 10.1093/schbul/sbx190 PMC6293230

[bibr86-23982128211009148] Robinson MB (1998) The family of sodium-dependent glutamate transporters: A focus on the GLT-1/EAAT2 subtype. Neurochemistry International 33(6): 479–491. 10098717 10.1016/s0197-0186(98)00055-2

[bibr87-23982128211009148] Ryan NM Lihm J Kramer M, et al. (2018) DNA sequence-level analyses reveal potential phenotypic modifiers in a large family with psychiatric disorders. Molecular Psychiatry 23(12): 2254–2265. 29880880 10.1038/s41380-018-0087-4 PMC6294736

[bibr88-23982128211009148] Sakai J (2020) Core Concept: How synaptic pruning shapes neural wiring during development and, possibly, in disease. Proceedings of the National Academy of Sciences of the United States of America 117(28): 16096–16099. 32581125 10.1073/pnas.2010281117 PMC7368197

[bibr89-23982128211009148] Samartzis L Dima D Fusar-Poli P, et al. (2014) White matter alterations in early stages of schizophrenia: A systematic review of diffusion tensor imaging studies. Journal of Neuroimaging 24(2): 101–110. 23317110 10.1111/j.1552-6569.2012.00779.x

[bibr90-23982128211009148] Sawa A Snyder SH (2005) Genetics. Two genes link two distinct psychoses. Science 310(5751): 1128–1129. 16293746 10.1126/science.1121114

[bibr91-23982128211009148] Sekar A Bialas AR de Rivera H, et al. (2016) Schizophrenia risk from complex variation of complement component 4. Nature 530(7589): 177–183. 26814963 10.1038/nature16549 PMC4752392

[bibr92-23982128211009148] Sellgren CM Gracias J Watmuff B, et al. (2019) Increased synapse elimination by microglia in schizophrenia patient-derived models of synaptic pruning. Nature Neuroscience 22(3): 374–385. 30718903 10.1038/s41593-018-0334-7 PMC6410571

[bibr93-23982128211009148] Shevelkin AV Terrillion CE Hasegawa Y, et al. (2020) Astrocyte DISC1 contributes to cognitive function in a brain region-dependent manner. Human Molecular Genetics 29(17): 2936–2950. 32803234 10.1093/hmg/ddaa180 PMC8248941

[bibr94-23982128211009148] Shimamoto C Ohnishi T Maekawa M, et al. (2014) Functional characterization of FABP3, 5 and 7 gene variants identified in schizophrenia and autism spectrum disorder and mouse behavioral studies. Human Molecular Genetics 23(24): 6495–6511. 25027319 10.1093/hmg/ddu369 PMC4240203

[bibr95-23982128211009148] Sommer IE Bearden CE Van Dellen E, et al. (2016) Early interventions in risk groups for schizophrenia: What are we waiting for? NPJ Schizophrenia 2: 16003. 10.1038/npjschz.2016.3 PMC4849435 27336054

[bibr96-23982128211009148] Steiner J Bernstein HG Bielau H, et al. (2008) S100B-immunopositive glia is elevated in paranoid as compared to residual schizophrenia: A morphometric study. Journal of Psychiatric Research 42(10): 868–876. 18001771 10.1016/j.jpsychires.2007.10.001

[bibr97-23982128211009148] Stevens B Allen NJ Vazquez LE, et al. (2007) The classical complement cascade mediates CNS synapse elimination. Cells 131(6): 1164–1178. 10.1016/j.cell.2007.10.036 18083105

[bibr98-23982128211009148] Tarasov VV Svistunov AA Chubarev VN, et al. (2019) Alterations of astrocytes in the context of schizophrenic dementia. Frontiers in Pharmacology 10: 1612. 10.3389/fphar.2019.01612 PMC7020441 32116664

[bibr99-23982128211009148] Terrillion CE Abazyan B Yang Z, et al. (2017) DISC1 in astrocytes influences adult neurogenesis and hippocampus-dependent behaviors in mice. Neuropsychopharmacology 42(11): 2242–2251. 28631721 10.1038/npp.2017.129 PMC5603806

[bibr100-23982128211009148] Toker L Mancarci BO Tripathy S, et al. (2018) Transcriptomic evidence for alterations in astrocytes and parvalbumin interneurons in subjects with bipolar disorder and schizophrenia. Biological Psychiatry 84(11): 787–796. 30177255 10.1016/j.biopsych.2018.07.010 PMC6226343

[bibr101-23982128211009148] Trépanier MO Hopperton KE Mizrahi R, et al. (2016) Postmortem evidence of cerebral inflammation in schizophrenia: A systematic review. Molecular Psychiatry 21(8): 1009–1026. 27271499 10.1038/mp.2016.90 PMC4960446

[bibr102-23982128211009148] Vainchtein ID Chin G Cho FS, et al. (2018) Astrocyte-derived interleukin-33 promotes microglial synapse engulfment and neural circuit development. Science 359(6381): 1269–1273. 29420261 10.1126/science.aal3589 PMC6070131

[bibr103-23982128211009148] Verkhratsky A Nedergaard M (2018) Physiology of astroglia. Physiological Reviews 98(1): 239–389. 29351512 10.1152/physrev.00042.2016 PMC6050349

[bibr104-23982128211009148] Walterfang M Wood SJ Velakoulis D, et al. (2005) Diseases of white matter and schizophrenia-like psychosis. Australian and New Zealand Journal of Psychiatry 39(9): 746–756. 10.1080/j.1440-1614.2005.01678.x 16168032

[bibr105-23982128211009148] Wang AM Pradhan S Coughlin JM, et al. (2019) Assessing brain metabolism with 7-T proton magnetic resonance spectroscopy in patients with first-episode psychosis. JAMA Psychiatry 76(3): 314–323. 30624573 10.1001/jamapsychiatry.2018.3637 PMC6439827

[bibr106-23982128211009148] Wang D Liu S Warrell J, et al. (2018) Comprehensive functional genomic resource and integrative model for the human brain. Science 362(6420): eaat8464. 10.1126/science.aat8464 PMC6413328 30545857

[bibr107-23982128211009148] Watanabe A Toyota T Owada Y, et al. (2007) Fabp7 maps to a quantitative trait locus for a schizophrenia endophenotype. PLOS Biology 5(11): e297. 10.1371/journal.pbio.0050297 PMC2071943 18001149

[bibr108-23982128211009148] Windrem MS Osipovitch M Liu Z, et al. (2017) Human iPSC glial mouse chimeras reveal glial contributions to schizophrenia. Cell Stem Cell 21(2): 195–208.e6. 10.1016/j.stem.2017.06.012 PMC5576346 28736215

[bibr109-23982128211009148] Yamasaki M Yamada K Furuya S, et al. (2001) 3-Phosphoglycerate dehydrogenase, a key enzyme for l-serine biosynthesis, is preferentially expressed in the radial glia/astrocyte lineage and olfactory ensheathing glia in the mouse brain. Journal of Neuroscience 21(19): 7691–7704. 11567059 10.1523/JNEUROSCI.21-19-07691.2001 PMC6762884

[bibr110-23982128211009148] Yang J Yang H Liu Y, et al. (2016) Astrocytes contribute to synapse elimination via type 2 inositol 1,4,5-trisphosphate receptor-dependent release of ATP. eLife 5: e15043. 10.7554/eLife.15043 PMC4829431 27067238

[bibr111-23982128211009148] Yang JH Wada A Yoshida K, et al. (2010) Brain-specific PHGDH deletion reveals a pivotal role for L-serine biosynthesis in controlling the level of D-serine, an N-methyl-D-aspartate receptor co-agonist, in adult brain. Journal of Biological Chemistry 285(53): 41380–41390. 10.1074/jbc.M110.187443 PMC3009864 20966073

[bibr112-23982128211009148] Zhang L Verwer RWH Lucassen PJ, et al. (2020) Prefrontal cortex alterations in glia gene expression in schizophrenia with and without suicide. Journal of Psychiatric Research 121: 31–38. 31739114 10.1016/j.jpsychires.2019.11.002

[bibr113-23982128211009148] Zhang Y Catts VS Sheedy D, et al. (2016) Cortical grey matter volume reduction in people with schizophrenia is associated with neuro-inflammation. Translational Psychiatry 6(12): e982. 10.1038/tp.2016.238 PMC5290336 27959331

